# 2816. Comparative Effectiveness of Meropenem/Vaborbactam vs. Ceftazidime/Avibactam among Adults Hospitalized with an Infectious Syndrome in the US, 2019-2021

**DOI:** 10.1093/ofid/ofad500.2427

**Published:** 2023-11-27

**Authors:** Marya D Zilberberg, Brian H Nathanson, Mark Redell, Kate Sulham, Andrew Shorr

**Affiliations:** EviMed Research Group, LLC, Goshen, Massachusetts; OptiStatim LLC, Longmeadow, Massachusetts; Melinta Therapeutics, Morristown, NJ; Melinta Therapeutics, Morristown, NJ; Medstar Washington Hospital Center, Not Applicable

## Abstract

**Background:**

Increasing rates of antimicrobial resistance have complicated the treatment of severe infections. Designated an “urgent threat” by the CDC, carbapenem-resistant Enterobacterales (CRE) lead to excess morbidity and mortality in multiple syndromes. Although several novel agents exist with activity against CRE, little data compares outcomes between them.

**Methods:**

To explore comparative effectiveness of meropenem/vaborbactam (MEV) vs. ceftazidime/avibactam (CZA), we conducted a multicenter retrospective cohort study within the Premier Healthcare Database, 2019-2021. We included all adult hospitalized patients who either were 1) admitted with or 2) during hospitalization had sepsis, a urinary tract (UTI), a complicated intraabdominal (cIAI) infection, or pneumonia. All syndromes were defined based on previously published administrative algorithms. We employed descriptive statistics to compare patients receiving MEV to those receiving CZA along demographic and clinical characteristics. We applied multiple regression models to adjust for confounding in the outcomes.

**Results:**

Among 1,989,765 patients who met enrollment criteria, 455 received MEV and 2,320 CZA. Compared to those on CZA, MEV patients were more likely to be Hispanic, more likely to suffer from diabetes and liver disease, but less likely to have chronic pulmonary disease (Table). They were more commonly seen in hospitals in the South and less so in teaching institutions. More MEV than CZA patients required dialysis sometime during hospitalization. Pneumonia as the index infection was less prevalent in the MEV than in the CZA group. In multivariable regression models adjusting for multiple confounders, patients on MEV had lower mortality and a reduced risk for ICU admission. We further observed a trend towards MEV-treated (vs. CZA-treated) patients having a shorter ICU length of stay (LOS) post-infection onset and a lower risk for incident *C. difficile* infection (Table).

Table

**Figure:**
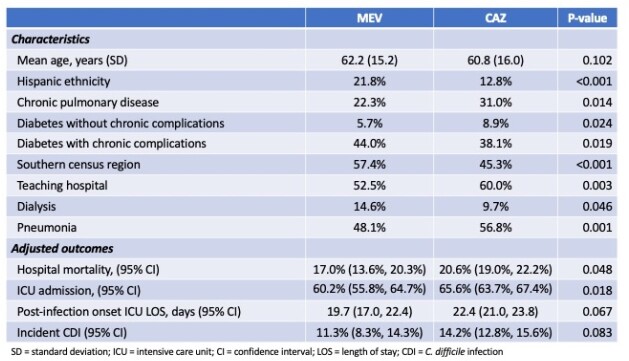

**Conclusion:**

In this cohort of hospitalized patients treated with either MEV or CZA for their infectious syndrome, MEV was associated with lower adjusted mortality and ICU admission, and a trend toward a reduction in incident CDI and ICU LOS post-infection onset compared to CZA.

**Disclosures:**

**Marya D. Zilberberg, MD, MPH**, Melinta Therapeutics: Grant/Research Support|Merck: Grant/Research Support|scPharmaceuticals: Advisor/Consultant|scPharmaceuticals: Grant/Research Support **Brian H. Nathanson, Ph.D.**, Merck & Co., Inc: Advisor/Consultant **Mark Redell, PharmD**, Melinta Therapeutics: Full-time employee|Melinta Therapeutics: Full-time employee|Melinta Therapeutics: Stocks/Bonds|Melinta Therapeutics: Stocks/Bonds **Kate Sulham, MPH**, Melinta Therapeutics: Advisor/Consultant

